# Vertical WS_2_/SnS_2_ van der Waals Heterostructure for Tunneling Transistors

**DOI:** 10.1038/s41598-018-35661-4

**Published:** 2018-12-10

**Authors:** Jiaxin Wang, Rundong Jia, Qianqian Huang, Chen Pan, Jiadi Zhu, Huimin Wang, Cheng Chen, Yawen Zhang, Yuchao Yang, Haisheng Song, Feng Miao, Ru Huang

**Affiliations:** 1 Key Laboratory of Microelectronic Devices and Circuits (MOE), Institute of Microelectronics, Peking University, Beijing, 100871 China; 2 School of Physics, Nanjing University, Nanjing, 210093 China; 3 Wuhan National Laboratory for Optoelectronics (WNLO), Huazhong University of Science and Technology, Wuhan, 430074 China

## Abstract

Van der Waals heterostructures composed of two-dimensional (2D) transition metal dichalcogenides (TMD) materials have stimulated tremendous research interest in various device applications, especially in energy-efficient future-generation electronics. Such ultra-thin stacks as tunnel junction theoretically present unprecedented possibilities of tunable relative band alignment and pristine interfaces, which enable significant performance enhancement for steep-slope tunneling transistors. In this work, the optimal 2D-2D heterostructure for tunneling transistors is presented and elaborately engineered, taking into consideration both electric properties and material stability. The key challenges, including band alignment and metal-to-2D semiconductor contact resistances, are optimized separately for integration. By using a new dry transfer technique for the vertical stack, the selected WS_2_/SnS_2_ heterostructure-based tunneling transistor is fabricated for the first time, and exhibits superior performance with comparable on-state current and steeper subthreshold slope than conventional FET, as well as on-off current ratio over 10^6^ which is among the highest value of 2D-2D tunneling transistors. A visible negative differential resistance feature is also observed. This work shows the great potential of 2D layered semiconductors for new heterostructure devices and can guide possible development of energy-efficient future-generation electronics.

## Introduction

Atomically thin two-dimensional (2D) semiconductors beyond graphene have emerged as one of the most promising material candidate for next generation electronic devices because of their sizable bandgap^1–6^. Recently, van der Waals heterostructures composed of 2D transition metal dichalcogenides (TMD) semiconductor with a broad range of material diversity have gained tremendous research interest in various device applications, especially in steep-slope tunneling transistors due to their superior properties beyond traditional bulk materials limits^1,2^. On one hand, van der Waals interactions enable the possibility for diverse heterostructures of highly distinct materials without the constraints of lattice matching, which is different from the covalent bonding in traditional bulk materials^1–4^. On the other hand, the dangling-bond-free interfaces of van der Waals heterostructure could mitigate the parasitic trap-assisted tunneling induced by interface states in traditional III-V-based tunneling heterojunction^1–7^. Moreover, for 2D-baesd tunneling heterojunctions, the relative band alignment can be modulated through electrostatic gating due to the van der Waals gap between the neighboring layers, theoretically suggesting that the type-II band alignment in the off-state could be modulated to type-III band alignment in the on-state for high drive current^8^. Consequently, tunneling transistors based on van der Waals heterostructures are expected to realize low leakage current, ultra-steep slope and high on/off current ratio simultaneously, which has been confirmed by lots of theoretical works and shows the great potential in low power electronics^9–12^. However, only a few experimental works have been reported regarding the tunneling transistors based on van der Waals heterostructures. Black Phosphorus (BP)/MoS_2_ tunneling transistors have been fabricated, while its high tunnel barrier (E_beff_) of approximately 0.5 eV at the heterojunction requires relatively large voltage to modulate the band alignment, demonstrating the limited on/off current ratio (~10^4^ at 3 V voltage range)^13^. Besides, BP material is unstable in the air, which would also cause the degradation of device performance^14–16^. Reported dual-gated tunneling transistors utilizing WSe_2_/MoS_2_ also exhibited the unsatisfactory on/off current ratio of 10^3^ due to the large E_beff_^8^. As an improvement, WSe_2_/SnSe_2_ tunneling transistors with E_beff_ lowered to 0.4 eV were reported and the on/off current ratio is remarkably enhanced^17^. However, SnSe_2_ is very unstable in the ambient environment, and can be easily oxidized^18^. Besides the E_beff_ and stability, the metal-to-2D semiconductor contact resistances would also severely limit the performance of 2D-based transistors^19^. Therefore, in spite of the optimism created by theoretical works, experimental optimization and demonstration of 2D-2D tunneling transistors with both high on/off current ratio and high material stability are still in urgent need.

In this work, the stable WS_2_/SnS_2_ van der Waals heterostructure with theoretically 0.02 eV E_beff_ is considered for the first time and selected as the optimal material platform for tunneling transistors. This optimal heterostructure is further experimentally demonstrated, and the WS_2_ and SnS_2_ serve as the p-type source layer and the n-type channel and drain layer, respectively. The key challenge of metal-to-2D semiconductor contact is further optimized for integration. Based on the physical insight into the metal/2D interfaces, work-function- and thickness- engineering are conducted for p-type WS_2_ and n-type SnS_2_ respectively to reduce the contact resistances. Based on a novel dry transfer technique for vertical heterostructure stack, the bottom-gated WS_2_/SnS_2_ tunneling transistor is fabricated and shows the on/off current ratio exceeding 10^6^, which is among the highest in the reported tunneling transistors. Compared with the conventional FET, comparable on-state current and steeper subthreshold slope (SS) are also obtained. The tunnel behaviors are further confirmed by low temperature measurements, and a visible negative differential resistance feature is observed. This work shows the great potential of van der Waals heterostructure for tunneling devices and future-generation energy-efficient electronics.

## Results and Discussion

**Figure 1:**
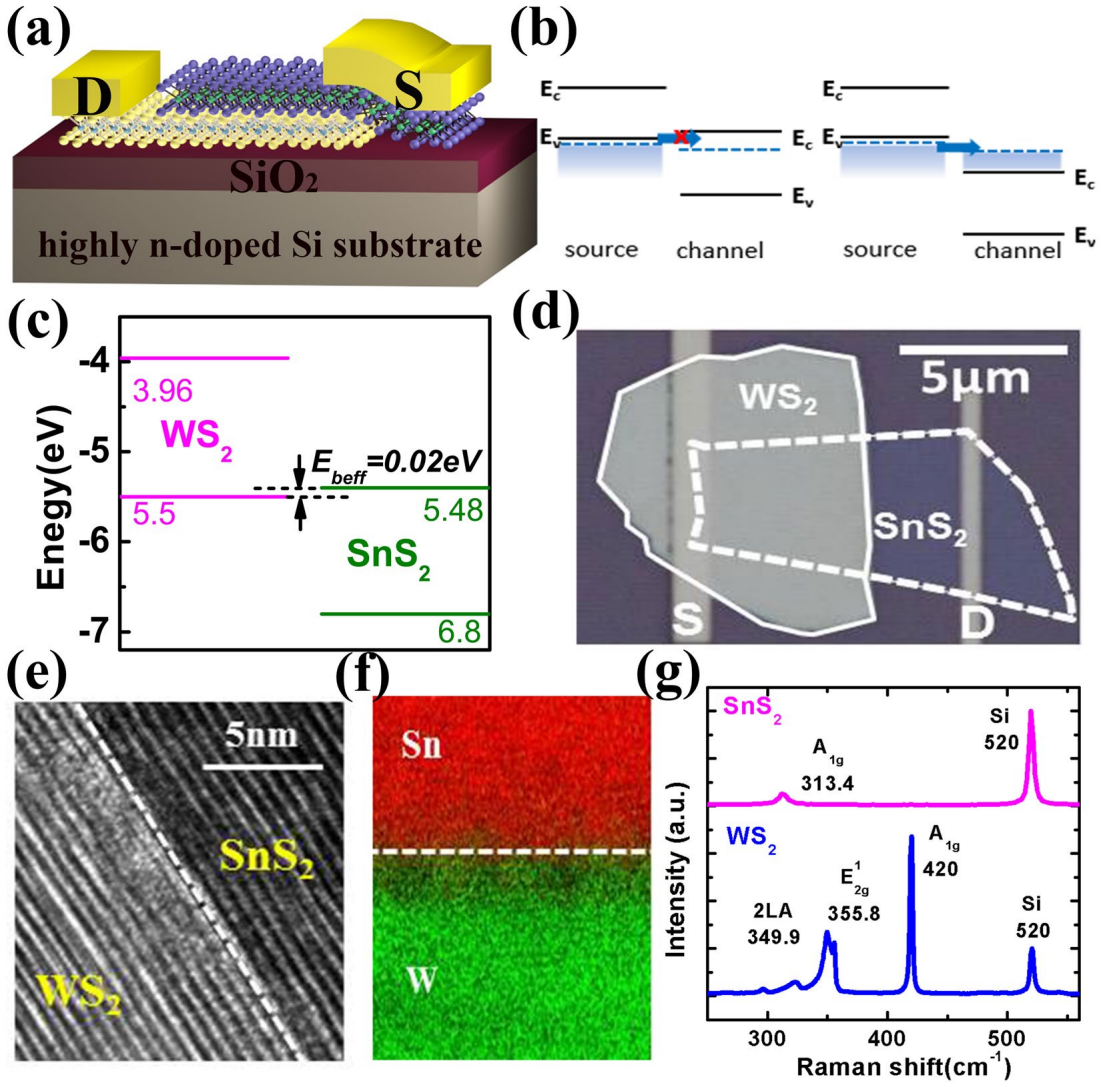
(**a**) Schematic view of the bottom-gated vertical tunneling transistor based on 2D semiconductors. Vertical tunneling occurs across the overlap region between p-type layer and n-type layer. (**b**) Band alignment in the off-state (left). There is no tunneling window between the valence band of the source layer and the conduction band of the channel layer. Band alignment in the on-state (right). The electric potential and carrier concentration of the channel layer are modulated by the bottom gate, and the tunneling window exists in which electrons can tunnel from the valence band of the source layer to the conduction band of the channel layer. (**c**) Band diagram of the WS_2_/SnS_2_ heterostructure with E_beff_ of 0.02 eV. (**d**) The optical microscope image of the fabricated n-type WS_2_/SnS_2_ tunneling transistor. (**e**) High-resolution STEM image and (**f**) EDS mapping of the WS_2_/SnS_2_ heterostructure. A clean and sharp interface is obtained. (**g**) Raman characterization of WS_2_ and SnS_2_ sheets used for tunneling transistors.

Figure 1a shows the schematic structure of bottom-gated vertical tunneling transistors based on the van der Waals heterostructure in this work. The SiO_2_ and highly n-doped Si are used as the gate dielectric and bottom gate, respectively. The channel layer is designed to be under the source layer, and the electric potential and carrier concentration of the channel layer are modulated by the bottom gate. The band alignment of the van der Waals heterostructure is designed to be type-II, in which the conduction band of the channel layer is above the valence band of the source layer. Figure 1b illustrates the operation mechanism of this n-type tunneling transistor. In the off-state, electrons in the valence band of the source layer cannot tunnel into the conduction band of the channel layer, since there is no tunneling window. As the bottom gate bias increases, the conduction band energy (E_C_) of the channel layer begins to be lower than the valence band energy (E_V_) of the source layer, and the tunnel current across the source layer/channel layer heterostructure will increase accordingly, exhibiting n-type characteristics. The band alignment can be tuned from type-II towards type-III due to the van der Waals gap, which would enhance the on/off current ratio of the transistor.

Taking into consideration of both device performance and material stability, the optimal heterostructure for tunneling transistor is selected by the following principles. First, from the perspective of on/off current ratio, the tunnel barrier E_beff_ should be considerably reduced so that low voltage is required to modulate the band alignment from type-II to type-III^17^. Second, tunneling electrons with smaller effective mass is beneficial for the higher tunnel efficiency and the higher tunnel current^20^. Third, according to our previous work, the lower density-of-state (DOS) of the channel layer would result in the better output characteristics of tunneling transistors with the smaller onset voltage and the better current saturation behavior^21^. At last, the stability of building materials to form heterostructures in ambient environment should also be taken into account for better device stability and reliability. Based on the above design rules, and according to the band structures of various 2D semiconductors from ab initio calculations^22,23^, p-WS_2_/n-SnS_2_ heterostructure stands out as the superior material platform for tunneling transistor. The E_beff_ in this near broken-gap heterostructure can be lowered to 0.02 eV theoretically, as shown in Fig. 1c. Meanwhile, the effective mass of electron in WS_2_ is relatively small compared to other metal dichalgonedies, and the low DOS of the channel layer SnS_2_ can improve the output characteristics of tunneling transistors in the meantime^24,25^. More importantly, WS_2_ and SnS_2_ is proved to possess great stability in ambient air^26,27^.

Figure 1d shows the optical image of a fabricated WS_2_/SnS_2_ tunneling transistor in this work. The bottom-gate dielectric of 300 nm-thick SiO_2_ was firstly grown on the highly n-doped Si substrate. Then, SnS_2_ and WS_2_ sheets were vertically stacked to form the van der Waals heterostructure, and after that, source and drain contacts were sequentially defined by electron beam lithography, electron beam evaporation and lift-off process.

For the heterostructure preparation, the SnS_2_ crystals were then grown by chemical vapor transport method and mechanically exfoliated onto the SiO_2_/Si substrate. The WS_2_ sheet was stacked upon SnS_2_ using a novel dry transfer process to avoid liquid contamination (see the dry transfer process in Supplementary Fig. S1), and the heterostructure was formed via van der Waals interaction. Figure 1e shows the high-resolution scanning TEM (STEM) image of the WS_2_/SnS_2_ heterostructure, confirming the clean interface obtained by the dry transfer process. The measured interlayer distance of WS_2_ is 0.66 nm and that of SnS_2_ is 0.6 nm, which agree well with values reported in refs ^28–30^. Energy-dispersive X-ray spectroscopy (EDS) composition analysis exhibits the sharp boundary between W and Sn, as shown in Fig. 1f. Raman spectra of these two sheets are shown in Fig. 1g and Raman peaks of both materials are clearly identified. The thicknesses of WS_2_ and SnS_2_ in this device are 23 nm and 4 nm, respectively. The characteristic Raman peaks of WS_2_ and SnS_2_ can be distinctly observed. WS_2_ shows the 2LA peak at 349.9 cm^−1^, E_2g_ peak at 355.8 cm^−1^, and A_1g_ peak at 420 cm^−1^. The A_1g_ peak of SnS_2_ is observed at 313.4 cm^−1^.

**Figure 2:**
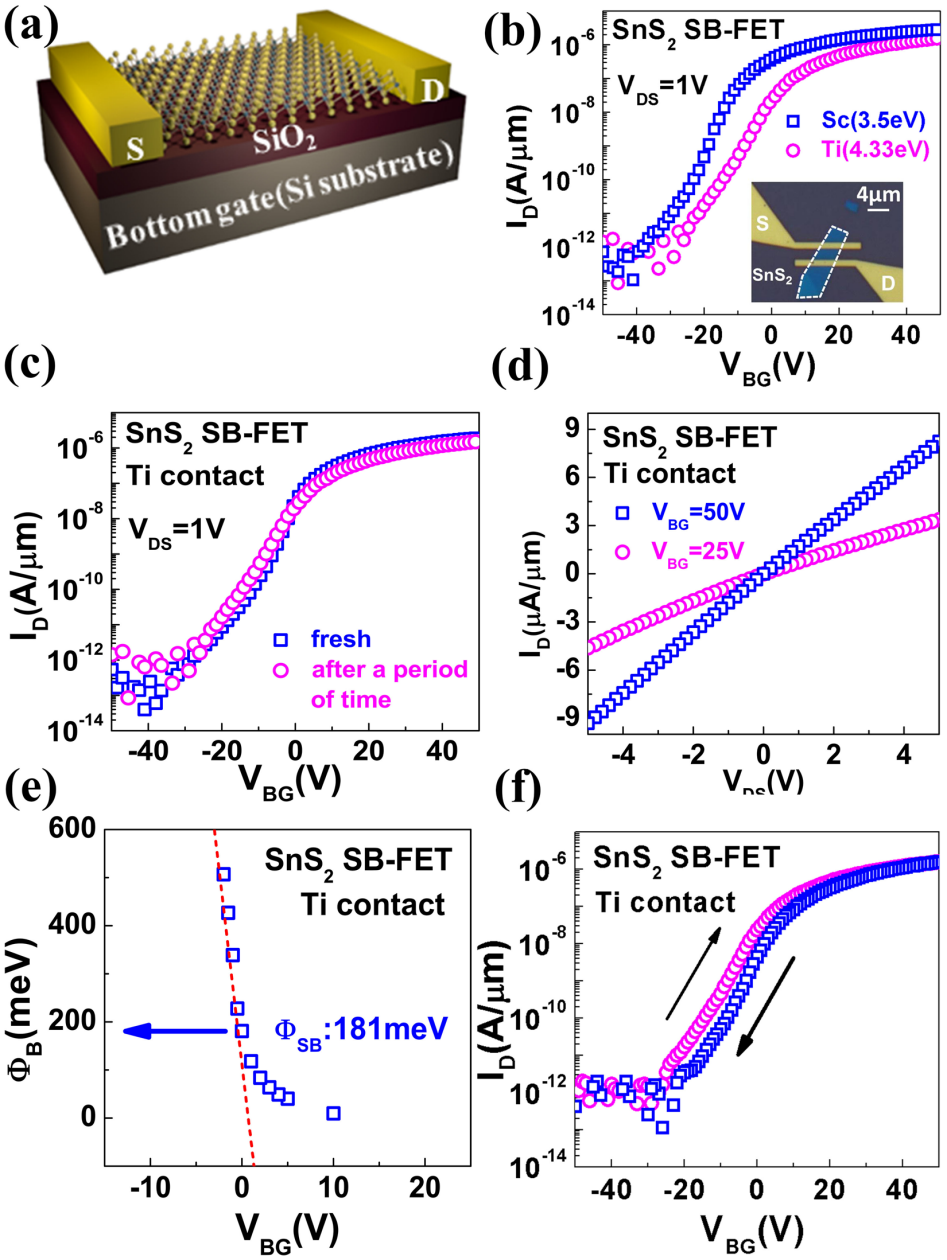
Optimization of n-type contact for SnS_2_. (**a**) Schematic view of bottom-gated SnS_2_ or WS_2_ SB-FETs. (**b**) Measured transfer characteristics of n-type SnS_2_ SB-FETs using Ti and Sc as contacts (V_BG_ is the voltage applied to the highly n-doped Si substrate). Inset is the optical microscope image of SnS_2_ SB-FET. (**c**) Transfer characteristics of SnS_2_ SB-FET with Ti contacts after a period of time, (**d**) Measured output characteristics of n-type SnS_2_ SB-FETs using Ti contacts. Linear dependence of I_D_ on V_DS_ indicates low contact resistance. (**e**) Extracted SB height for the Ti-to-SnS_2_ contact. (**f**) Measured hysteresis characteristics of the SnS_2_ SB-FET with Ti contacts, showing no significant hysteresis.

In order to reduce the contact resistances for better device performance, contact-engineering is conducted for p-type WS_2_ and n-type SnS_2_, separately. To date, contacts of metal-to-2D semiconductors in the majority of reported metal dichalcogenide transistors are Schottky contacts instead of Ohmic contacts due to the difficulty of heavy doping in thin 2D semiconductors, which is also the case in WS_2_ and SnS_2_^28,31–33^. In principle, low work functions (W_m_) of contact metals lead to the small Schottky barrier (SB) height for electrons, and high work function metals are beneficial for low hole barriers. In order to investigate the SB height at metal-to-2D semiconductor contacts, bottom-gated SnS_2_ and WS_2_ SB-FETs were fabricated and characterized firstly as shown in Fig. 2a. For SnS_2_ SB-FETs, Ti (W_m_ = 4.33 eV) and Sc (W_m_ = 3.5 eV) were adopted for realizing n-type contacts due to their low W_m_^34^. Both Ti- and Sc-contacted SnS_2_ SB-FETs exhibit n-type transistor behaviors (Fig. 2b). Yet compared with Ti, Sc would be easily oxidized in the air, resulting in severe degradation of transfer characteristics for Sc-contacted SB-FETs over time (Fig. 2c and Supplementary Fig. S2). Therefore, Ti with the better stability is chosen as the electrode for contacts to SnS_2_ in the n-type tunneling transistors in this work. Figure 2d shows the measured output characteristics of Ti-contacted SB-FETs, and the linear dependence of current on drain voltage further confirms the low contact resistance at Ti/SnS_2_ interfaces^33^. The extracted Schottky barrier height is as low as 0.181 eV (Fig. 2e), and the detailed extraction method can be seen in Supplementary Fig. S3. Addtionally, no significant hysteresis is observed in the transfer characteristics of SnS_2_ FET with Ti contacts (Fig. 2f).

**Figure 3:**
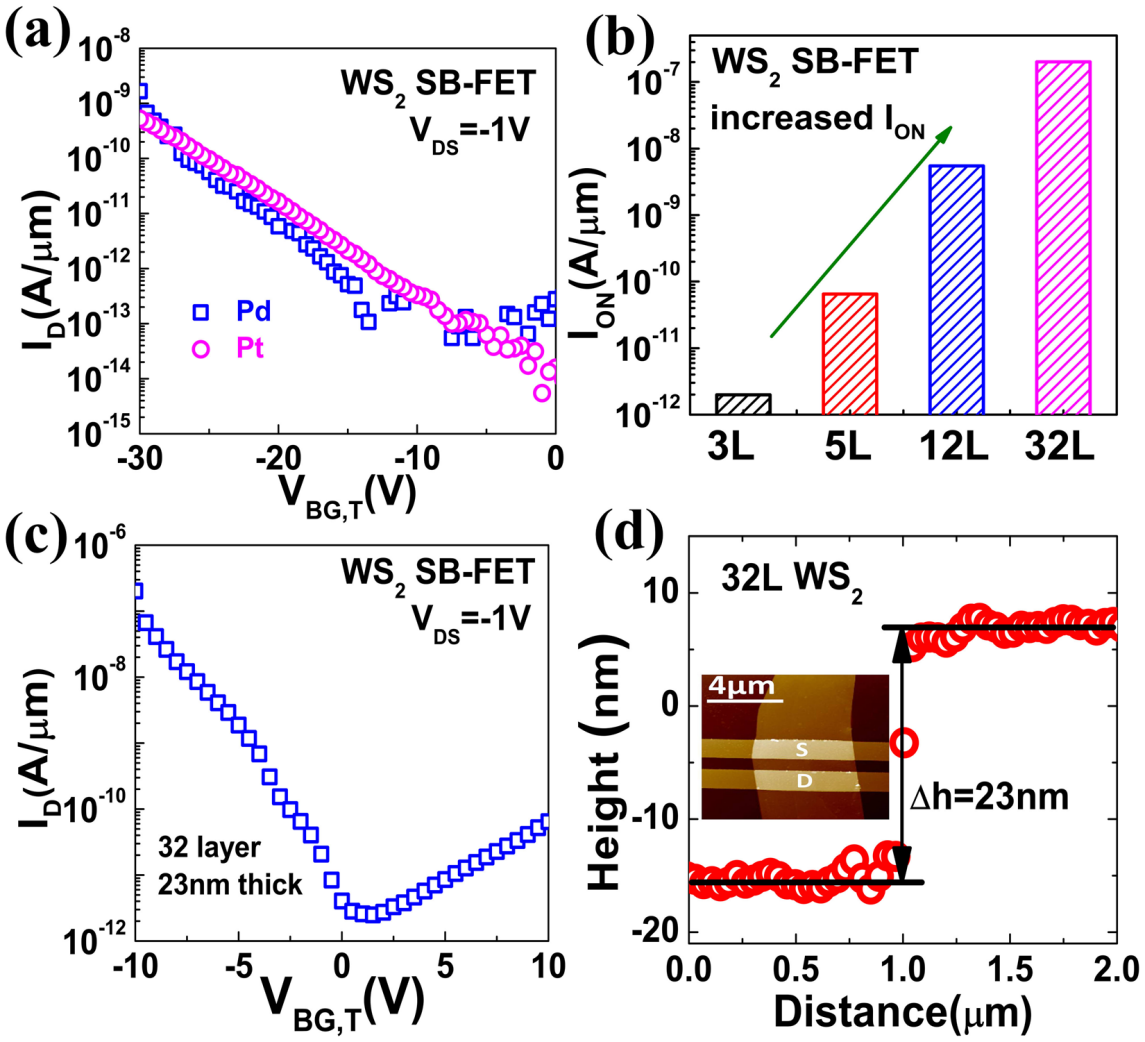
Optimization of p-type contact for WS_2_. (**a**) Measured transfer characteristics of p-type WS_2_ SB-FETs using Pd and Pt contacts (V_BG,T_ = V_BG_ − V_onset(Pt)_, V_onset(Pt)_: the onset voltage at which hole current begins to increase in the Pt-contacted SB-FET). Similar currents are observed in these two devices. (**b**) Dependence of on-state current density on WS_2_ thickness in WS_2_ SB-FETs. The on-state hole current increases with the number of WS_2_ layers. (**c**) Measured transfer characteristics of SB-FET with 23 nm-thick WS_2_. Large hole current in the on-state indicates the low resistance p-type contact. (**d**) AFM height profile of the 32-layer WS_2_ sheet. Inset is the corresponding AFM image of fabricated devices.

Apart from n-type contacts to SnS_2_, the resistance of p-type contacts to WS_2_ was also investigated. High work function metals, Pd (W_m_ = 5.12 eV) and Pt (W_m_ = 5.65 eV), were chosen as the metal electrodes to realize low-resistance p-type contacts^34^. Figure 3a shows the measured transfer characteristics of WS_2_ SB-FETs. Although there is a significant difference (0.53 eV) of work functions between Pd and Pt, hole current in the Pd-contacted SB-FET is comparable to that in Pt-contacted device, indicating the strong Fermi-level pinning at the metal/WS_2_ interface. In order to suppress Fermi-level pinning at the metal-to-2D material interface, inserting a thin layer of substoichiometric molybdenum trioxide (MoOx) between metal and 2D materials has been verified as an effective way to facilitate hole injection, which can be attributed to the high work function of MoO_x_ and its excellent interface properties with 2D materials^31^. However, the work function of MoO_x_ is very sensitive to ambient gas exposure, and thus the device performance will degrade in the air. Therefore, a novel approach is proposed in this work to reduce the SB height for holes at metal-to-WS_2_ contacts. As we know, the bandgaps of 2D semiconductors are thickness-sensitive due to the influence of quantum confinement effect^23^. According to results from ab initio calculations, as the thickness of WS_2_ layers increases, the valence band moves upward while the position of conduction band remains nearly unchanged^23^. As a consequence, the SB height for holes can be decreased with thicker WS_2_. In this work, SB-FETs with different numbers of WS_2_ layers were fabricated and Fig. 3b shows the corresponding hole currents. As the number of WS_2_ layers increases from 3 to 12, hole currents are increased by three decades. When the thickness of WS_2_ layers is increased to 23 nm, WS_2_ p-type SB-FET exhibits high I_ON_ exceeding 0.2 μA μm^−1^ (Fig. 3c), suggesting low resistance of fabricated p-type contacts. Figure 3d shows the atomic force microscope (AFM) image of the 23 nm-thick WS_2_ SB-FET, and the height difference between the WS_2_ sheet and the substrate.

**Figure 4:**
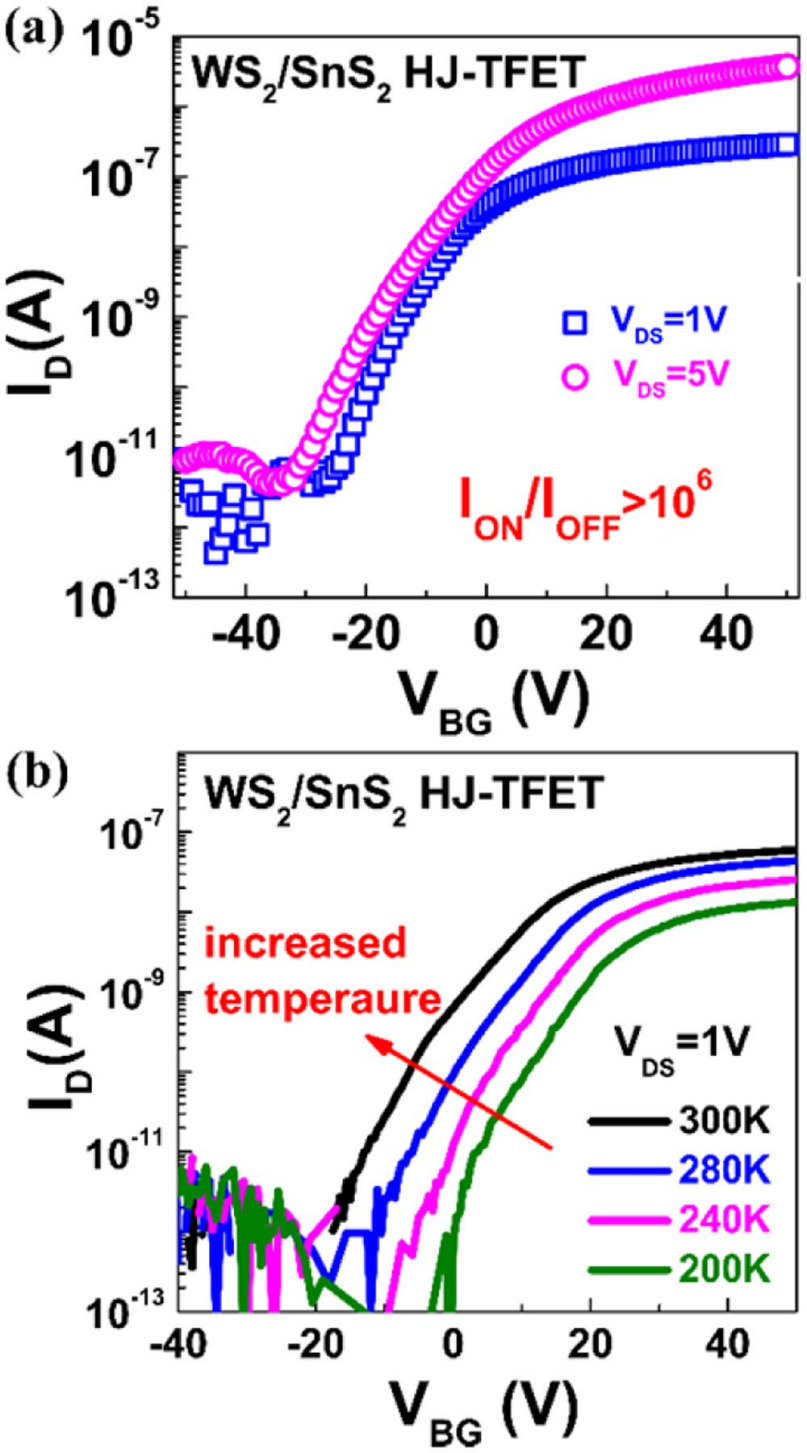
Electrical characteristics of the WS_2_/SnS_2_ tunneling transistor. (**a**) Measured transfer characteristics of the bottom-gated WS_2_/SnS_2_ tunneling transistor. The on-state current (I_ON_) is 3.7 μA with an area of 20 μm^2^ and on/off current ratio is over 10^6^. (**b**) Measured temperature characteristics of the bottom-gated WS_2_/SnS_2_ tunneling transistor.

Based on the optimized n-type and p-type contacts, the tunneling transistors based on WS_2_/SnS_2_ van der Waals heterostructures are experimentally demonstrated with Pt as the source contact and Ti as the drain contact, and electrical characterization at different temperatures and under different bias conditions are performed. The thicknesses of the WS_2_ flakes are designed according to the above contact optimization, and are measured to be 23 nm by AFM. In contrast, the contact resistance of n-type metal-to-SnS_2_ is thicknesses insensitive due to the nearly unchanged position of conduction band when layer number increases, and the SnS_2_ used in tunneling transistor is measured to be 4 nm. Figure 4a shows the measured typical transfer characteristics of the n-type WS_2_/SnS_2_ tunneling transistor at room temperature. The results are obtained by applying the bias on SnS_2_ contact, with WS_2_ contact grounded. With the optimized design of metal-to-2D semiconductor contacts, the n-type WS_2_/SnS_2_ tunneling transistor exhibits high I_ON_ of 3.7 μA. The on/off current ratio of the fabricated device is over 10^6^ and the on-state current density is 186 nA μm^−2^. The high on/off current ratio, and high on-state current which is comparable with the value obtained in the SnS_2_ SB-FET, further confirm the optimized band alignment in WS_2_/SnS_2_ tunneling transistors. Compared with conventional SB-FET in Fig. 2b, the subthreshold slope of the fabricated WS_2_/SnS_2_ device is much steeper and also increases with gate voltage which is a typical feature of tunnel transistors. Since the transistor is fabricated based on bottom-gated structure with 300 nm-thick SiO_2_, the value of SS is relatively large, and can be further optimized by reducing the gate oxide thickness or incorporating with high-κ dielectrics. In order to further verify the BTBT mechanism of this n-type transistor, the dependence of transfer characteristics on temperature is studied. As shown in Fig. 4b, I_ON_ shows the positive dependence on temperature and SS changes little with temperature, exhibiting the typical features of BTBT operation mechanism^35–38^. The weak dependence of SS on temperature also indicates the suppression of trap-assisted tunneling, which benefits from the clean interface obtained from the dry transfer process.

**Figure 5:**
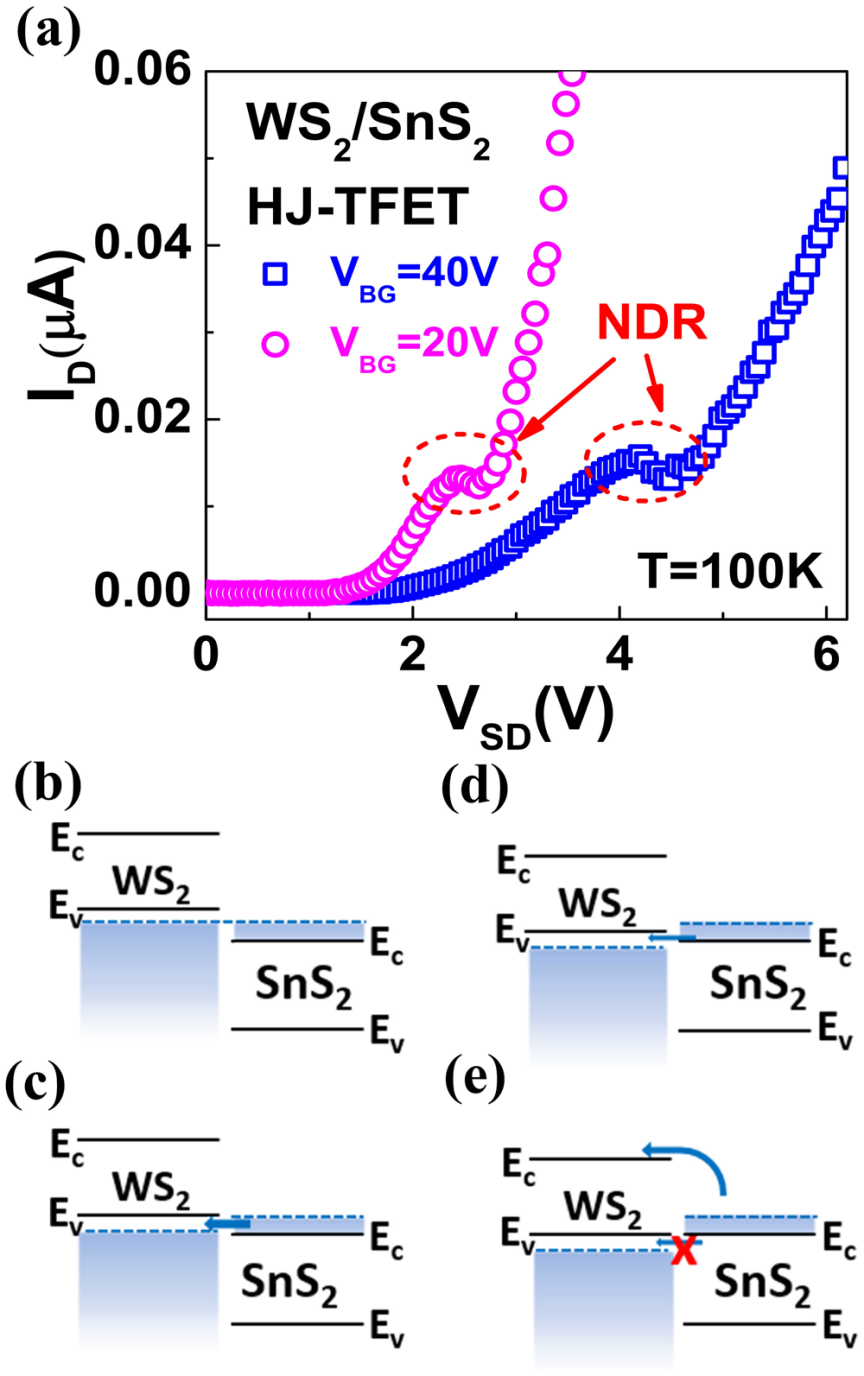
Negative differential resistance (NDR) feature of the WS_2_/SnS_2_ tunneling transistor. (**a**) Measured output characteristics of the bottom-gated WS_2_/SnS_2_ tunneling transistor in the forward bias region. Clear NDR is observed for positive gate voltages. (**b**) Band alignment at V_SD_ = 0 V. Fermi level in WS_2_ and SnS_2_ is aligned. (**c**) Band alignment at V_SD_ > 0 V. The band of p-type WS_2_ is pulled down and a finite tunneling window is created for electrons in the conduction band of SnS_2_ to tunnel into the empty states in the valence band of WS_2_. The tunnel current reaches its peak when the Fermi level of WS_2_ aligns with the conduction band minimum of SnS_2_. With further increasing V_SD_ (**d**), the tunnel window is gradually switched off and the reduction of tunnel current leads to NDR. When V_SD_ continues to increase (**e**), the thermal injection current dominates and increases with the reduced energy barrier.

In order to further validate the BTBT mechanism in this WS_2_/SnS_2_ heterostructure, the output characteristics in forward bias region are investigated. Figure 5a shows the output characteristics at 100 K, which are measured by applying the voltage on WS_2_ contact, with SnS_2_ contact grounded. The distinct negative differential resistance (NDR) is observed and confirms that a heavily-doped p-n junction is formed at the interface due to charge transfer^17^. The band alignments of the WS_2_/SnS_2_ herterostructure under different bias conditions are illustrated in Fig. 5b–e. In the equilibrium state, the Fermi level in p-WS_2_ and n-SnS_2_ is aligned, as shown in Fig. 5b. As the source-drain voltage V_SD_ increases, the energy band of WS_2_ is pulled down and a finite tunneling window is created for electrons in the conduction band of SnS_2_ to tunnel into the empty states in the valence band of WS_2_. The tunnel current reaches its peak when the Fermi level of WS_2_ aligns with the conduction band minimum of SnS_2_, as shown in Fig. 5c. With V_SD_ further increasing, the tunneling window is gradually switched off and the reduction of tunnel current leads to NDR (Fig. 5d). After that, thermal injection current begins to dominate the total current due to the reduced thermal barrier, and increases with V_SD_ (Fig. 5e). It should be noted that the peak voltage can be modulated by the bottom-gate voltage V_BG_ in this device. The conduction band of SnS_2_ varies with V_BG_, and the peak voltage needed to align the Fermi level of WS_2_ with the conduction band of SnS_2_ changes consequently.

## Conclusions

In conclusion, the optimal WS_2_/SnS_2_ van der Waals heterostructure for tunneling transistors is presented and elaborately engineered, taking into consideration both electric properties and material stability. Besides, the key challenge of metal-to-2D semiconductor contact is optimized to achieve low-resistance n-type and p-type contacts for SnS_2_ and WS_2_, respectively. Ti contact with low work function and superior stability can induce small Schottky barrier height for electrons at metal-to-SnS_2_ contacts. Low-resistance p-type contacts are obtained at the metal/WS_2_ interface through the thickness optimization of WS_2_. With the optimized metal-to-2D semiconductor contacts and a proposed new dry transfer technique for vertical heterostructure stack, the fabricated n-type WS_2_/SnS_2_ tunneling transistor exhibits superior performance with the high on/off current ratio over 10^6^, as well as comparable on-state current and steeper subthreshold slope compared with conventional FET, showing the great potential of van der Waals heterostructure for future energy-efficient devices.

## Methods

### Device Fabrication

The highly n-type Si substrate with 300 nm thermal silicon oxide is prepared as the bottom gate structure. The starting materials used for the fabrication of n-type WS_2_/SnS_2_ tunneling transistors were high-quality bulk crystals of WS_2_ and SnS_2_. The process flow of the bottom-gated WS_2_/SnS_2_ tunneling transistor has been described in the Supplementary Fig. S1. In details, 10 nm Ti/20 nm Au was deposited to form the Ti-to-SnS_2_ contact, and the Pt-to-WS_2_ contact was generated with 20 nm Pt/40 nm Au.

### Physical Characterization

AFM and Raman spectra were used to characterize the thicknesses of WS_2_ and SnS_2_. Raman spectra were excited by 514 nm laser with the spot diameter about 1 μm. The laser power was kept less than 0.1 mW to avoid sample heating and oxidation in the air. Structural characterization by scanning TEM (STEM) was performed in JEM-ARM200F with the acceleration voltage of 100 keV. The STEM sample was prepared by focused ion beam (FIB) using the gallium beam.

### Electrical Measurements

N-type WS_2_/SnS_2_ tunneling transistors were electrically characterized in the vacuum chamber using the Agilent B1500A semiconductor parameter analyzer.

## Electronic supplementary material

Supplementary Information

## Data Availability

All data supporting this study and its findings are available within the article, its Supplementary Information and associated files. Any source data deemed relevant is available from the corresponding author upon request.
