# Supplementary material for: Vertical WS2/SnS2 van der Waals Heterostructure for Tunneling Transistors
Source: Sci Rep. 2018 Dec 10;8:17755. doi: 10.1038/s41598-018-35661-4 (PMC6288168; doi:10.1038/s41598-018-35661-4)
Supplement: Supplementary file 1 — Supplementary Information [file 41598_2018_35661_MOESM1_ESM.pdf]

Supplementary Information for

Vertical WS_2/SnS_2 van der Waals Heterostructure for Tunneling Transistors

Jiaxin Wang[†1], Rundong Jia[†1], Qianqian Huang[1*], Chen Pan[2], Jiadi Zhu[1], Huimin Wang[1], Cheng Chen[1], Yawen Zhang[1], Yuchao Yang[1], Haisheng Song[3], Feng Miao[2] and Ru Huang[1*]

[1]*Key Laboratory of Microelectronic Devices and Circuits (MOE), Institute of Microelectronics, Peking University, Beijing 100871, China.*

[2]*School of Physics, Nanjing University, Nanjing 210093, China.*

[3]*Wuhan National Laboratory for Optoelectronics (WNLO), Huazhong University of Science and Technology, Wuhan 430074, China.*

[†]*Jiaxin Wang and Rundong Jia contributed equally to this work.*

[*]Correspondence should be addressed to: ruhuang@pku.edu.cn, hqq@pku.edu.cn

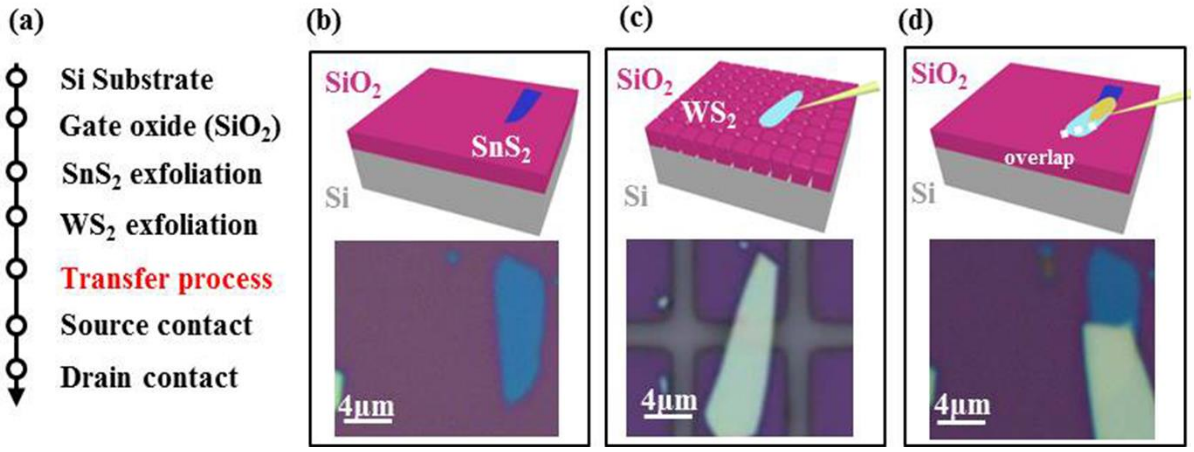

Supplementary Figure 1. The novel dry transfer process used to fabricate WS_2/SnS_2 tunneling transistors. (a) Experimental process flow of bottom-gated WS_2/SnS_2 tunneling transistors. **(b)** Schematic illustration (up) and corresponding optical microscope images (down) of the exfoliated SnS_2 sheet. **(c)** Schematic illustration (up) and corresponding optical microscope images (down) of the exfoliated WS_2 sheet. **(d)** Schematic illustration (up) and corresponding optical microscope images (down) of the WS_2/SnS_2 heterostructures obtained by our dry transfer process. In detail, a glass needle, which could be precisely controlled by micromanipulators under the optical microscope, was brought into the trench in SiO_2 and pick up the WS_2 layer exfoliated on SiO_2. After aligning the WS_2 with the target SnS_2 sheet, heterostructure was formed via van der Waals adhesion.

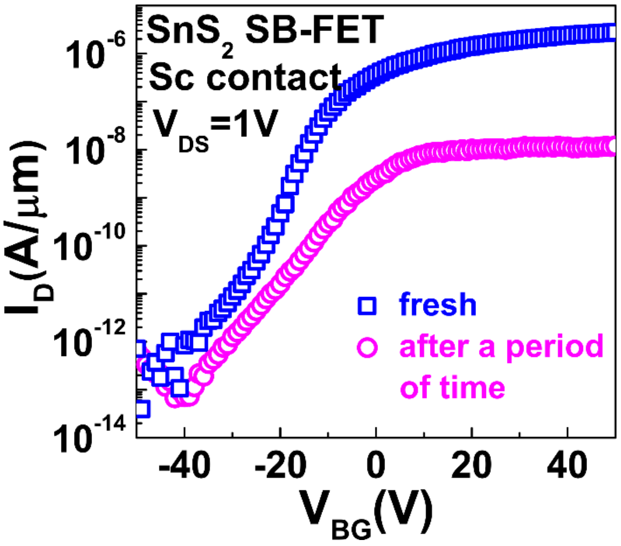

Supplementary Figure 2. Measured degradation of transfer characteristics for SnS_2 SB-FET with Sc contacts.

Fig. S2 illustrates that the transfer characteristics of SnS_2 SB-FET with Sc contacts degrade considerably after a period of time, while the SnS_2 SB-FET with Ti contacts is more stable over time even without the passivation layer on it, as shown in Fig. 2c (in the main article).

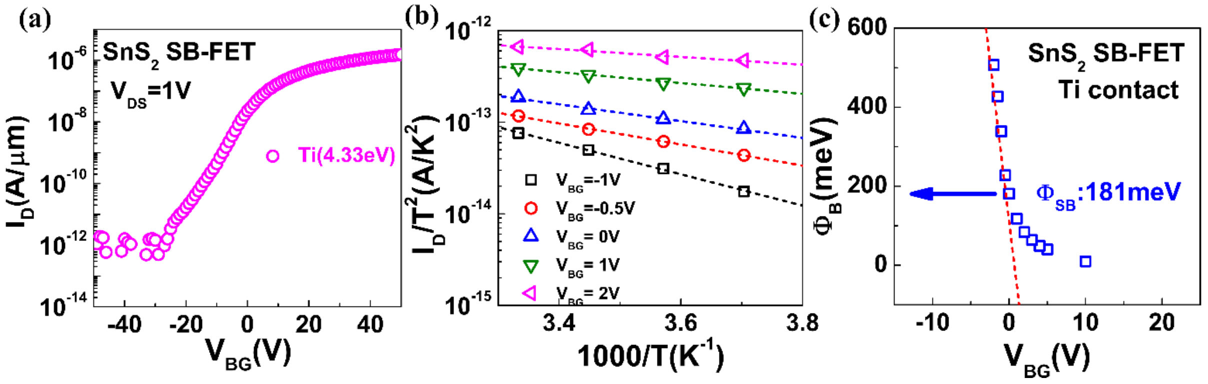

Supplementary Figure 3. Extraction of Schottky barrier (SB) height of SnS_2 SB-FET. (a) Measured transfer characteristics of the SnS_2 SB-FET with Ti contacts. **(b)** Arrhenius plot of the SnS_2 SB-FET with Ti contacts. **(c)** Extracted SB height for the Ti-to-SnS_2 contact.

In the n-type SnS_2 SB-FET, the Schottky Barrier (SB) heights are extracted on the basis of thermionic emission theory. The drain current is formed by electron injection through reversed-biased source Schottky junction, including the thermal emission current and thermally assisted tunneling current. When the gate voltage is smaller than the flat band voltage (V_{FB}), the thermal emission current dominates and the drain current can be expressed in the following equation:

$$I_D = AA^*T^2 \exp\left(-\frac{q\Phi_B}{kT}\right)\left[1-\exp\left(-\frac{qV_{DS}}{kT}\right)\right]$$

Where, A is the area of Schottky junction, A^* is the Richardson's constant, k is the Boltzmann constant, q is the electronic charge, T is the temperature, V_{DS} is the drain to source bias. Φ_B is the barrier height that electrons has to overcome, and the Schottky barrier height can be denoted as Φ_{SB}. As shown in Fig. S3b, Φ_B can be extracted from the relationship:

$$\log\left(\frac{I_D}{T^2}\right) \propto -\frac{q\Phi_B}{kT}$$

The extracted Φ_B is linearly dependent on the bottom-gate voltage for smaller V_{BG}. When the gate voltage equals to V_{FB}, the extracted Φ_B corresponds to Φ_{SB}.

As the gate voltage increases beyond V_{FB}, the thermally assisted tunneling current begins to increase, and the relationship between Φ_B and V_{BG} deviates from linearity. As illustrated in Fig. S3c, the Schottky barrier height Φ_{SB} for the Ti-to-SnS_2 contact is determined to be 181 meV.
